# Nepafenac-Loaded Cyclodextrin/Polymer Nanoaggregates: A New Approach to Eye Drop Formulation

**DOI:** 10.3390/ma12020229

**Published:** 2019-01-11

**Authors:** Blanca Lorenzo-Veiga, Hakon Hrafn Sigurdsson, Thorsteinn Loftsson

**Affiliations:** Faculty of Pharmaceutical Sciences, University of Iceland, Hofsvallagata 53, IS-107 Reykjavik, Iceland; blv3@hi.is (B.L.-V.); thorstlo@hi.is (T.L.)

**Keywords:** cyclodextrin, nepafenac, polymer, complexation, aggregate, self-assemble, ocular drug delivery

## Abstract

The topical administration route is commonly used for targeting therapeutics to the eye; however, improving the bioavailability of drugs applied directly to the eye remains a challenge. Different strategies have been studied to address this challenge. One of them is the use of aggregates that are formed easily by self-assembly of cyclodextrin (CD)/drug complexes in aqueous solution. The aim of this study was to design a new eye drop formulation based on aggregates formed between CD/drug complexes. For this purpose, the physicochemical properties of the aggregates associated with six CDs and selected water-soluble polymers were analysed. Complex formation was studied using differential scanning calorimetry (DSC), Fourier-transform infrared spectroscopy (FT-IR) and ^1^H nuclear magnetic resonance spectroscopy (^1^H-NMR). Results showed that HPβCD performed best in terms of solubilization, while γCD performed best in terms of enhancing nanoaggregate formation. Formation of inclusion complexes was confirmed by DSC, FT-IR and ^1^H-NMR studies. A mixture of 15% (*w*/*v*) γCD and 8% (*w*/*v*) HPβCD was selected for formulation studies. It was concluded that formulations with aggregate sizes less than 1 µm and viscosity around 10–19 centipoises can be easily prepared using a mixture of CDs. Formulations containing polymeric drug/CD nanoaggregates represent an interesting strategy for enhanced topical delivery of nepafenac.

## 1. Introduction

Nepafenac (2-amino-3-benzoylbenzeneacetamide) is a potent non-steroidal anti-inflammatory drug (NSAID) used to treat the pain and inflammation associated with cataract surgery. It is currently available as a 0.1% ophthalmic suspension. However, in powdered form, nepafenac is known to have low water solubility and low tissue permeability and is classified as a class IV compound by the Biopharmaceutical Classification System [1,2,3,4]. Thus, developing a new eye drop formulation of nepafenac with improved bioavailability is of considerable interest.

Improving the bioavailability of a drug applied topically to the eye remains a challenge [5,6,7]. Different strategies to improve bioavailability include the use of penetration enhancers [8], viscosity modifiers [9], carrier systems or external forces such as electrical currents or ultrasounds and drug/cyclodextrin (CD) complexation [10,11,12,13].

CDs are cyclic oligosaccharides of α-D-glucopyranose that contain a hydrophobic central cavity and have a hydrophilic outer surface. The natural CDs, α, β and γ, are composed of six, seven or eight D-glucopyranose units linked by α1, 4 glycosidic bonds (see Supplementary Information, Figure S1). CDs have been widely explored in polymer chemistry because of their ability to form complexes with hydrophilic polymers, monomers and drugs. Chemically crosslinked or grafted with polymer, CDs have been proposed for use in smart drug delivery systems in many studies [10,11,14,15,16,17,18].

Native or non-substituted CDs and their hydroxy-propyl derivatives have been used as pharmaceutical excipients to increase drug solubility, improve chemical stability, reduce toxicity and transport molecules to specific sites [19,20,21,22,23]. Importantly, drug/CD complexation increases the aqueous solubility of poorly soluble drugs without altering their properties [24]; as a result, CDs have been used in over 40 marketed products to date. Nevertheless, solubility enhancement via drug/CD complexation has certain limitations such as the high molecular weight of the CDs, toxicity issues and high costs [25,26,27].

The addition of a second solubilizing agent to a drug/CD complex to form a ternary system has been reported as an interesting strategy [21,28,29,30,31,32,33]. For example, the incorporation of salts [34], co-solvents [35], amino acids [36] or hydrophilic polymers [37,38,39] in the complexation media can improve the solubility of the drug and make the formulation more cost effective by allowing use of lower concentrations of CDs. Mennini et al. [31] studied the effect of an amino acid, L-arginine, in enhancing the complexation and solubilizing abilities of randomly-methylated-βCD (RameβCD) toward oxaprozin. They found that complexion with RameβCD and simultaneous salt formation with L-arginine was a successful strategy for improving the solubility and dissolution properties of oxaprozin. Soliman et al. [40] explored the effect of different hydrophilic polymers including polyethylene glycol (PEG-4000), chitosan, polyvinyl pirrolidine (PVP K-30), hydroxypropylmethyl cellulose (HPMC) and hydroxyethyl cellulose (HEC), on avanafil/β-CD inclusion complexes. They confirmed that the addition of 7% PVP K-30 to avanafil/β-CD inclusion complexes improved complex stability. Also, they found that using higher concentrations of some of these polymers (PVP K-30 or PEG-4000) led to a decrease in avanafil solubility, suggesting that they can displace the drug from the CD cavity at high concentrations. The use of supramolecular structures formed between CDs and amphiphilic copolymers, known as poly(pseudo)rotaxanes, have been extensively investigated [11,41,42].

Eye drop formulations designed by our group containing CDs have been shown to deliver lipophilic drugs effectively both to the anterior and posterior segment of the eye, despite the various ocular barriers that make delivering new drug formulations to the eye a challenge [43,44,45,46]. The aim of this study was to formulate a new aqueous-based anti-inflammatory eye drop containing nepafenac, CD and polymer. For this purpose, different studies were performed: (1) to evaluate the physicochemical characteristics of the nepafenac/CD complex, (2) to study the influence of selected hydrophilic polymers on the solubility of the nepafenac/CD complex, (3) to investigate the possible synergistic effect of using a mixture of γCD and HPβCD on complex solubility and (4) to characterize the solid-state inclusion complex.

## 2. Materials and Methods

### 2.1. Materials

Nepafenac was purchased from Sigma-Aldrich (St. Louis, MO, USA). α-Cyclodextrin (αCD), β-cyclodextrin (βCD) and γ-cyclodextrin (γCD) were obtained from Wacker Chemie (Munich, Germany). 2-Hydroxypropyl-α- cyclodextrin (HPαCD), 2-hydroxypropyl-β-cyclodextrin (HPβCD) and 2-hydroxypropyl-γ-cyclodextrin (HPγCD) were kindly donated by Janssen Pharmaceutica (Beerse, Belgium).

Polyvinylpyrrolidone (PVP) (average MW 10.000 kDa), 87–90% hydrolysed poly(vinyl alcohol) (PVA) (average MW 30.000–70.000 kDa), carboxymethylcellulose (CMC) sodium salt (low viscosity), hydroxypropylmethylcellulose (HPMC; viscosity approx. 100 centipoises) and reagent grade tyloxapol were obtained from Sigma-Aldrich (St. Louis, MO, USA). Methyl cellulose (MC; viscosity approx. 15 centipoises) was purchased from ICN Biomedicals Inc. (Solon, OH, USA). Membrane filters (0.45 µm) were purchased from Phenomenex (Cheshire, UK).

All other chemicals used were of analytical reagent grade purity. Milli-Q (Millipore, Billerica, MA, USA) water was used for the preparation of all solutions.

### 2.2. Moisture Content of CDs

A small amount (1 g) of solid powdered γCD and HPβCD were placed in separate aluminium pans and their water content measured using an A&D MX-50 moisture analyser (A&D company, Limited, Tokyo, Japan). Measurements were made in triplicate. The water content of γCD and HPβCD was 10.70% and 6.22%, respectively.

### 2.3. Chemical Stability of Nepafenac

The chemical stability of nepafenac was determined in aqueous solution containing 1% *w*/*v* γCD following heating by sonication [47,48,49,50]. The solution was shaken for 24 h until the drug was completely dissolved and then passed through a 0.45 µm membrane filter. The solution was then divided between four sealed vials. Vial 1 was used as a blank. Vials 2, 3 and 4 were heated in a sonicator at 60 °C for 20, 40 and 60 min, respectively. Drug concentrations were determined by HPLC.

### 2.4. Quantitative Analysis

The HPLC assay was performed using a reverse-phase ultra-high-performance liquid chromatography (UHPLC) Ultimate 3000 series system (Dionex Softron GmbH, Germering, Germany) consisting of a LPG-3400SD pump (Dionex, Germering, Germany) with a built-in degasser, WPS-3000 autosampler (Dionex, Germering, Germany), TCC-3100 column compartment (Dionex, Germering, Germany) and CoronaR ultra RS detector (Dionex, Germering, Germany). During the stationary phase, a Phenomenex Kinetex C18 column (150 × 4.6 mm, 5 µm) with matching HPLC Security Guard (Phenomenex, Cheshire, UK) was used. The mobile phase used a mixture of acetonitrile and water (50:50). The flow rate was 1 mL/min, the column oven temperature was ±25 °C and the detection wave length was set to 254 nm. The retention time for nepafenac under these conditions was 2.3 min.

Peak area and other variables were analysed using the software Chromeleon version 7.2 SR4 (ThermoScientific, Waltham, MA, USA).

### 2.5. Phase Solubility Studies

**Figure 1:**
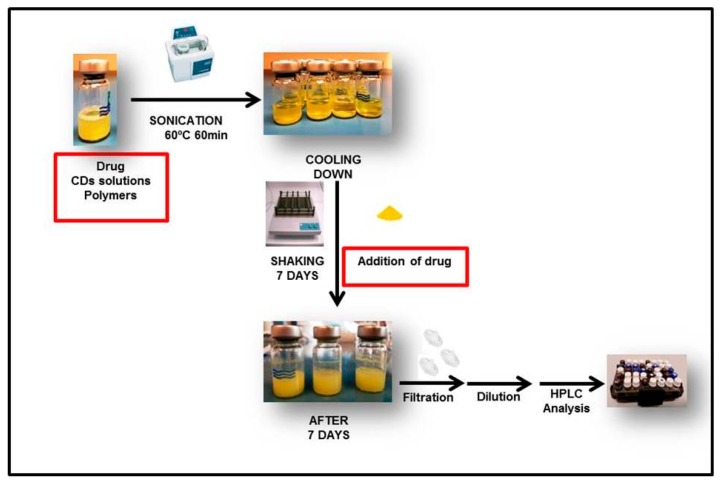
Schematic representation describing the steps involved in the formation of drug/CD inclusion complexes in phase solubility studies.

The solubility of nepafenac in combination with different cyclodextrin concentrations was determined following heating by sonication [47,49,50]. The schematic representation of this method is shown in Figure 1. Firstly, an excess amount of nepafenac (approximately 5 mg) was added to aqueous solutions containing known concentrations of CDs (ranging up to 15% (*w*/*v*) for αCD, γCD, HPαCD, HPβCD, HPγCD and 1.5% (*w*/*v*) for βCD) in pure water. The drug suspensions were saturated with nepafenac and heated in a sonicator in sealed vials at 60 °C for 60 min, before being allowed to cool to room temperature. Then, a small amount of solid nepafenac (approximately 2 mg) was added to each suspension to produce drug precipitation. Vials were resealed and placed in a shaker under constant agitation for 7 days. After reaching equilibrium, suspensions were filtered using 0.45 µm membrane filters, before being diluted in pure water and analysed by UHPLC. Determinations were made in triplicate.

The most frequent method to study the formation of complexes is through the phase solubility studies proposed by Higuchi and Connor. In them, we can distinguish different solubility profiles (type A and B) depending on the effect of the cyclodextrin on the solubilization of the drug [51].

The apparent stability constant (K_1:1_) according to the hypothesis of a 1:1 stoichiometric ratio of complexes was calculated using the following equation:

(1) $$K_{1:1}\;=\;\frac{\mathrm{slope}}{S_{0}\left(1-\mathrm{slope}\right)}$$

In addition, complexation efficiency (CE) was calculated. This factor can be calculated either from the slope of the phase solubility profile or from the ratio of the concentration of the drug/CD complex to free CD [52]:

(2) $$\mathrm{CE}=\frac{\mathrm{slope}}{\left(1-\mathrm{slope}\right)}=\frac{[\mathrm{Guess}/\mathrm{CD}\;\mathrm{complex}]}{\left[\mathrm{CD}\right]}$$

### 2.6. Complex Characterization in Solid State

#### 2.6.1. Preparation of Inclusion Complexes

Samples were prepared using a freeze drying method [53,54]. Clear supernatant solutions from phase solubility studies of γCD and HPβCD that had shown Al-type profiles were used to confirm the presence of nepafenac/CDs complexes. 200 µL was collected from each vial, placed in small Eppendorfs and freeze-dried at −55 °C for 24 h in a Snijders scientific 2040 Freeze dryer (Snijders Labs, Tilburg, The Netherlands).

#### 2.6.2. Fourier Transform Infra-Red (FT-IR) Spectroscopy

The FT-IR spectra of pure nepafenac, pure CDs and their freeze-dried complexes were measured with a FT-IR spectrometer (Thermo Fisher Scientific model Nicolet iS10, Waltham, MA, USA) using an Attenuated Total Reflectance (ATR) technique. Data were obtained in the range of 500–4000 cm^−1^. Analyses were performed at room temperature.

#### 2.6.3. Differential Scanning Calorimetry (DSC)

DSC curves were recorded on Netzsch DSC 214 polyma (Netzsch Group, Selb, Germany). Samples (approximately 3–5 mg) were heated at the rate of 10 °C/min in sealed aluminium pans under nitrogen. The temperature ranged from 30 to 250 °C. An empty aluminium pan was used as a reference.

### 2.7. Structure of Inclusion Complexes Combining Nepafenac with γCD and HPβCD

^1^H-NMR spectrums were analysed to study inclusion complexes. Experiments were carried out at 500 MHz in a Brucker AVANCE 400 instrument (Bruker Biospin GmbH, Rheinstetten, Germany). Deuterated chloroform (CDCl_3_-d_6_) was used to dissolve nepafenac and deuterium oxide (D_2_O) to dissolve nepafenac/CD complexes, γCD and HPβCD.

### 2.8. Influence of Water-Soluble Polymers on Solubility of Complexes and Effect of Mixtures of γCD and HPβCD

PVP, PVA, CMC and tyloxapol were selected as polymers. The polymer was firstly dissolved in pure water and then added to aqueous solutions containing CD to a final concentration of 1% *w*/*v*. The solubility of nepafenac was analysed by UHPLC method previously validated in Section 2.4. Effect of cyclodextrins and excipients on osmolality, viscosity and size of binary and ternary systems with nepafenac were also analysed (see Supplementary Information, Table S1). All samples were prepared in triplicate.

### 2.9. Preparation and Characterization of 0.5% (w/v) Nepafenac Eye Drops

Nine formulations were prepared (Table 1) and all of them contained: 0.5% (*w*/*v*) nepafenac, 15% (*w*/*v*) γCD, 8% (*w*/*v*) HPβCD, 0.1% (*w*/*v*) EDTA, 0.02% (*w*/*v*) benzalkonium chloride, 0.05% (*w*/*v*) sodium chloride and different ratios of polymers as outlined in Table 1.

#### 2.9.1. Solid Drug Fraction

The formulation (6 mL) being tested was centrifuged at 13,000 rpm (MC6 centrifuge, Sarstedt AG, Nümbrecht, Germany) at room temperature (22–23 °C) for 30 min and the supernatant was analysed by HPLC. The drug content in solid phase was calculated as:

(3) $$\%\;\mathrm{solid}\;\mathrm{drug}\;\mathrm{fraction}\;(\mathrm{SDF})=\frac{\left(\mathrm{total}\;\mathrm{drug}-\mathrm{dissolved}\;\mathrm{drug}\right)}{\mathrm{Total}\;\mathrm{drug}\;\mathrm{content}}\times 100$$

#### 2.9.2. Dynamic Light Scattering

The particle sizes within the eye drop formulations were characterized by dynamic light scattering using a Nanotrac Wave particle size analyser from Microtrac Inc. (Montgomeryville, PA, USA). Measurements were in triplicate as described previously.

#### 2.9.3. Physicochemical Properties

The viscosity of the eye drop formulations was measured using a Brookfield viscometer (model DV2T) attached to a Brookfield water bath (model TC-150) with a spindle (CPA-40Z) operating at 25 °C (Middleborough, MA, USA). Each formulation was measured in triplicate. The osmolality of the formulations was determined using an Osmomat 030 Gonotec (Berlin, Germany) freezing point osmometer.

#### 2.9.4. Transmission Electron Microscope (TEM) Analysis

The morphology of nepafenac-loaded CD/polymer nanoaggregates was studied visually by TEM. Samples were prepared using 4% of uranyl acetate as negative staining agent. Firstly, 3 µL of each sample was loaded into a coated grid in a parafilm^®^ located inside a petri dish and left to dry for 30 min at 37–40 °C. After centrifugation of uranyl acetate at 10,000 rpm for 5 min, a drop of 26 µL of the dye was transferred to another petri dish containing a parafilm^®^ flip-loaded grid onto uranyl acetate and left for 5 min. Finally, the excess of dye was removed and the grid dried with filter paper and left at room temperature during 12 h. Finally, the samples were analysed using a Model JEM 1400 TEM (JEOL, Tokyo, Japan).

## 3. Results and Discussion

### 3.1. Stability of Nepafenac in Autoclave and Sonicator

The chemical stability of nepafenac in CD aqueous solutions after heating in sonicator was studied (Table 2). From the results, can be seen that nepafenac/CD complexes could be prepared using sonication as a heating method for the phase-solubility studies since it was safe, ease to use and no degradation of nepafenac was observed.

### 3.2. Phase-Solubility Studies

**Figure 2:**
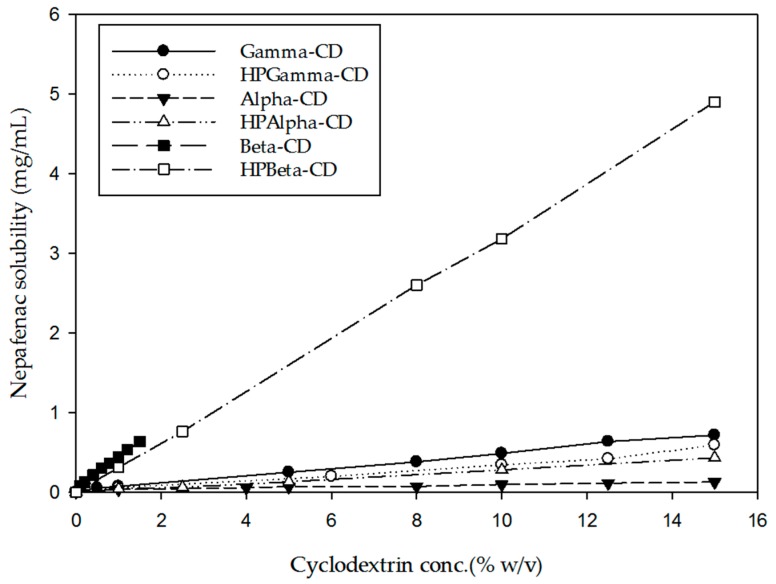
Phase-solubility profile of nepafenac in aqueous solution in combination with different CDs. Results are expressed as mean (n = 3).

The solubility of nepafenac in water in the presence of the different CD forms can be seen in Table 3. Phase-solubility profiles of nepafenac in aqueous CD solutions containing αCD, βCD, γCD, HPαCD, HPβCD and HPγCD are shown in Figure 2. Based on the phase-solubility profiles, the solubility of nepafenac increases with increasing CD concentration in the aqueous media.

According to the Higuchi–Connors classification system, inclusion complexes for all CDs studied were soluble. Complexes including γCD, βCD, αCD, HPβCD, HPαCD showed an A_l_ profile, indicating that the solubility of the drug increased linearly with increasing CD concentration. However, HPγCD showed an A_p_-type profile, indicating a positive deviation from linearity. The presence of an Al profile with a slope less than 1, suggested that a 1:1 nepafenac/CD complex has been formed.

Among the different CDs investigated, the highest CE was found for HPβCD. Challa et al. [55] have previously recommended the CDs γCD, HPβCD and SBEβCD for use in ocular drug delivery. Moreover, studies on CD toxicity in a human cornea epithelial cell line by Saarinen-Savolainen et al. [56] revealed that γCD had the least cytotoxic profile, followed jointly by HPβCD and SBEβCD, then DMβCD and finally αCD. Johannsdottir et al. [45] demonstrated that γCD also had the highest capacity compared with αCD for forming nanoparticles in aqueous solution that were able to solubilize hydrophobic drugs. Based on the phase solubility profiles obtained in our study and the safety profile and capacity of CDs to form nanoparticles reported in previous studies, mixtures of γCD and HPβCD were selected for further study.

### 3.3. Influence of Adding Water-Soluble Polymers on the Solubility of Nepafanec/CD Complexes and Impact of Mixing γCD and HPβCD

**Figure 3:**
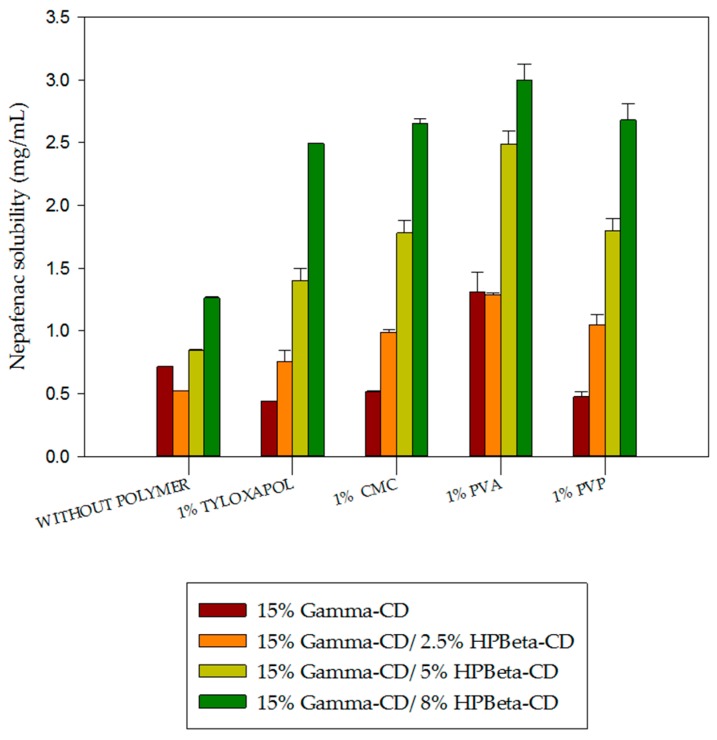
Impact of cyclodextrins and excipients on the solubility of binary and ternary complexes including nepafenac.

The impact of adding PVP, PVA, CMC and tyloxapol on the solubility of nepafenac in pure γCD and mixed γCD/HPβCD solutions is shown in Figure 3. Tyloxapol is a non-ionic polymer with surfactant properties. PVA, PVP and CMC are also polymers known as “viscosity modifiers”. All of them are widely used in the preparation of eye drops.

In Figure 3, results showed that the addition of 1% PVP, CMC and tyloxapol had a slightly negative effect on nepafenac/γCD complex solubility. However, nepafenac/γCD complex solubility was almost tripled following the addition of 1% PVA. PVA was also associated with higher solubility than other polymers when added to nepafenac/CD complexes containing a mixture of γCD and HPβCD. No synergistic effect was found by combining 15% γCD with 2.5% HPβCD (the solubility of nepafenac in pure 2.5% HPβCD is 0.85 mg/mL—see Figure 2). However, when we carried out the solid state characterization of the complex, its formation was easily achieved using higher amounts of HPβCD, such as 8%. The impact of mixing CDs on solubility was previously investigated by Jansook et al. [57]. This group studied the synergistic effect between γCD and HPγCD using a variety of drugs, including dexamethasone [58]. They found that synergistic-type effects only occurred when a drug with a B-type profile was combined with γCD and HPγCD. This may explain why no synergistic effect was found when combining the A-type profile drug nepafenac with γCD or HPβCD.

### 3.4. Solid State Characterization of Nepafenac/CD Inclusion Complexes

#### 3.4.1. FT-IR Spectra

**Figure 4:**
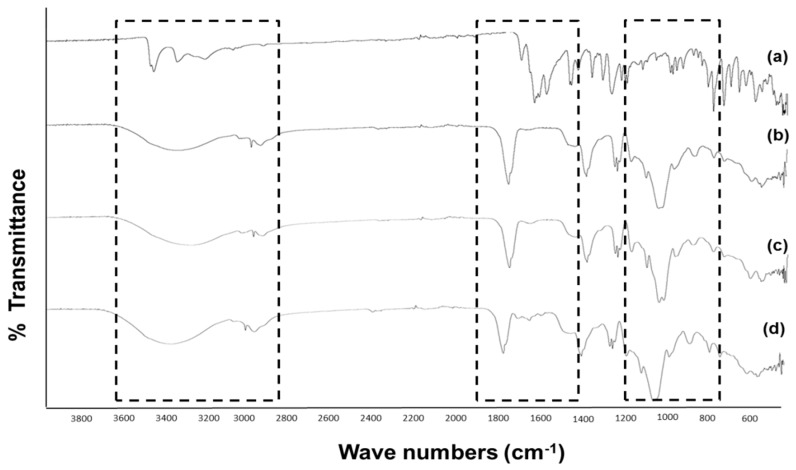
Comparison of FT-IR spectra of: (**a**) pure nepafenac, (**b**) pure HPβCD, (**c**) pure γCD and (**d**) freeze-dried nepafenac/15%γCD/8%HPβCD complex.

Fourier transform infra-red (FT-IR) spectroscopy was applied to confirm the presence of guest and host molecules in the inclusion complex. The FT-IR spectra of pure nepafenac, γCD and HPβCD, as well as nepafenac/CD complexes prepared by freeze-drying are shown in Figure 4. Bands representing pure compounds were compared to the band for the complex. The disappearance or change in position of peaks indicates the formation of complexes.

For pure nepafenac, characteristic absorption peaks appeared at 1631 cm^−1^ (attributed to C=O stretch absorption of the secondary amide group), 1664 cm^−1^ (attributed to C=O stretch absorption of the ketone group), 3500–3300 cm^−1^ (attributed to NH_2_ stretch absorption) and 3080, 3040, 1968 and 1818 cm^−1^ (attributed to benzene aromatic stretching) (Figure 4a). For pure γCD and HPβCD (Figure 4b,c, respectively), the characteristic absorption bands relating to OH stretch were observed at 3300, 3410, 1420 and 1330 cm^−1^, while the absorption bands relating to CO stretch were seen at 1079 and 1029 cm^−1^. In the case of freeze-dried nepafenac/γCD/HPβCD complex (Figure 4d), the NH_2_ stretch and benzene aromatic stretch absorption bands were less intense than for pure nepafenac, suggesting that this part of the nepafenac compound may be encapsulated within the complex containing both CDs.

#### 3.4.2. DSC

**Figure 5:**
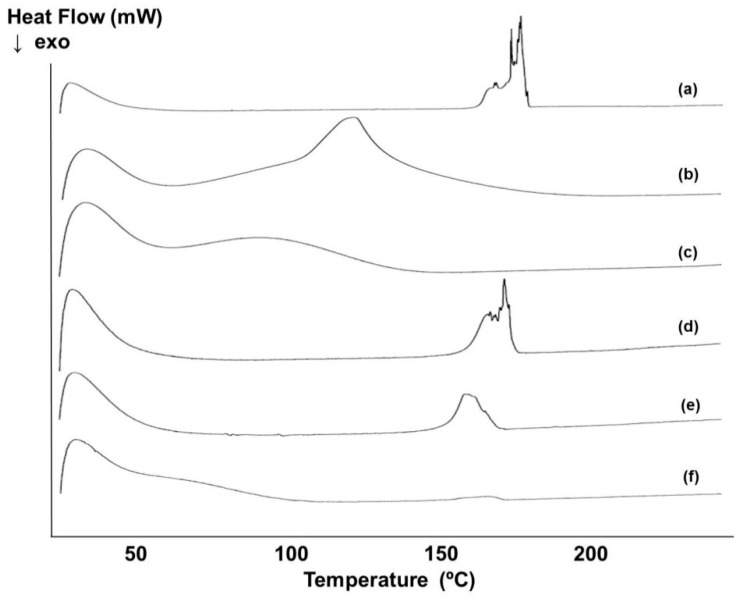
Differential scanning calorimetry (DSC) curves for: (**a**) pure nepafenac, (**b**) pure γCD, (**c**) pure HPβCD, (**d**) freeze-dried nepafenac and mixture of 15%γCD/2.5%HPβCD complex, (**e**) freeze-dried of nepafenac/15%γCD/5%HPβCD complex and (**f**) freeze-dried nepafenac/15%γCD/8%HPβCD complex. Exo; exothermic.

DSC measurements were used to obtain information about the thermal stability and phase transition of all components. This thermal method confirmed the solid-state interaction between nepafenac and both CDs since their DSC curves (Figure 5d–f) showed shifting to lower temperatures than the melting point of nepafenac (Figure 5a). The DSC curves for nepafenac, CDs and their complexes are presented in Figure 5.

The DSC curve for pure nepafenac showed a sharp endothermic peak at 178 °C, corresponding to the melting point of the drug (Figure 5a). DSC curves for γCD and HPβCD showed a wide endothermal effect between approximately 30 °C and 150 °C (Figure 5b,c), as a result of the dehydration process. For the freeze-dried ternary complex (nepafenac/15%γCD/2.5%HPβCD) (Figure 5d), the intensity of the endothermic peak was reduced and also shifted to lower temperatures compared with nepafenac. A similar pattern was seen for the nepafenac/15%γCD/5%HPβCD complex relative to the 15%γCD/2.5%HPβCD complex, with both a decrease in the intensity of the endothermic peak and a shift to a lower temperature range. This noticeable decrease in intensity of the endothermic peak and shift to a lower temperature is indicative of a loss of nepafenac crystalline structure and the formation of a solid dispersion. Moreover, for the nepafenac/15%γCD/8%HPβCD complex, the endothermic peak corresponding to the melting point of the drug vanished. This may be due to the formation of solid-state nepafenac /CD inclusion complexes.

### 3.5. Structure of Nepafenac/γCD/HPβCD Inclusion Complexes

^1^H-NMR spectroscopy has become the most important method for structural elucidation of organic compounds in solution state [59]. These studies provide useful information on the characteristics of guest/host CD inclusion complexes, including the orientation of the guest molecule inside the hydrophobic cavity of the CD host molecule [60,61]. The formation of inclusion complexes lead to chemical shifts (Δδ) in the ^1^H-NMR spectra of the guest molecule; Δδ can be calculated using the following equation:

(4) $$\Delta \delta^{*}=\delta_{\mathrm{complex}}-\delta_{\mathrm{free}}$$

where δ_complex_ and δ_free_ are chemical shifts between free and bound CD molecules, respectively. Chemical shifts are shown in ppm.

**Figure 6:**
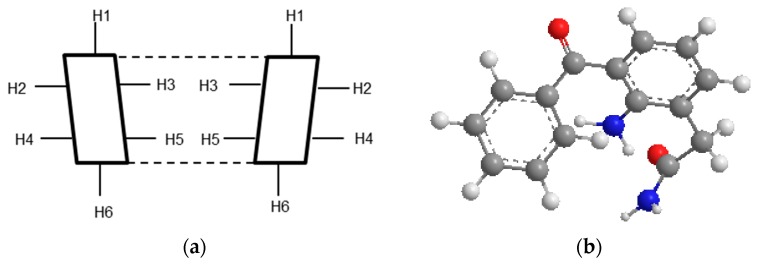
Cross-section of CD molecule (**a**) and nepafenac 3D structure (**b**).

^1^H-NMR spectroscopy has proven useful in the study of the formation of CD inclusion complexes with many compounds [62,63]. In γCD, there are six protons: the H-3 and H-5 protons are located inside the cavity, whereas the others (H-1, H-2, H-4 and H-6) are located on the exterior of the CD molecule (Figure 6). In the case of HPβCD, there is an additional methyl group [64]. In order to confirm the formation of the inclusion complex of nepafenac with γ- and HPβ-CD, a one-dimensional ^1^H NMR study was performed (see Supplementary Information, Figure S2). The difference in the chemical shifts between free and bound CDs molecules are shown in Table 4 and Table 5.

The changes in Δδ of γCD in the presence of nepafenac for the H-3 (+0.0218) and H-5 (+0.0213) protons were downfield (Table 4). Moreover, the Δδ of the H-3 proton was higher than that for the H-5 proton. These results showed that the guest molecule, nepafenac, occupies the entire volume of the cavity inside γCD.

As shown in Table 5, changes in Δδ for the H-3 (−0.034) and H-5 (−0.1329) proton of HPβCD were upfield, indicating the formation of an inclusion complex. Also, Δδ* of the H-5 proton was higher than Δδ* of the H-3 proton, suggesting partial inclusion of nepafenac in the HPβCD cavity [64,65].

2D correlation spectroscopy was conducted to confirm the location of the guest in the complex. Chemical shifts corresponding to free nepafenac alone and in the presence of γCD or HPβCD were also examined (See Supplementary Information, Table S2).

### 3.6. Characterization of Formulation of 0.5% (w/v) Nepafenac Eye Drops

As shown in Table 6, the formulations with the largest drug solubilization capacity—F3, F4 and F1—had the highest recorded solid drug fraction values (63.6%, 62.4% and 61.3%, respectively). Several parameters can affect the viscosity of eye drops, such as the addition of surfactants, ions and also particle size. The formulations with the highest viscosity levels were F2, F3 and F5, with values between 14 and 19 centipoises (cP), making them suitable for use as eye drops. The formulations F8 and F1 had viscosity levels of approximately 4 cP and so were not considered for the formulation of eye drops due to low viscosity. The addition of 1% (*w*/*v*) CMC in formulations F2, F3, F5 and F7 led to higher viscosity and osmolality values than observed for the formulations that did not contain this polymer. Aggregates were between 208 and 581 nm in diameter for all formulations, with the exception of F1 and F8 which had diameters of 98 and 17 nm, respectively.

The best results in terms of solubility, size and viscosity were obtained for formulation F3 which contains 2% (*w*/*v*) PVA, 1% (*w*/*v*) CMC and 0.1% (*w*/*v*) tyloxapol. In all cases, more sodium chloride (NaCl) should be added to reach normal osmolality values of about 300 mOsm/kg [45].

#### TEM Analysis

**Figure 7:**
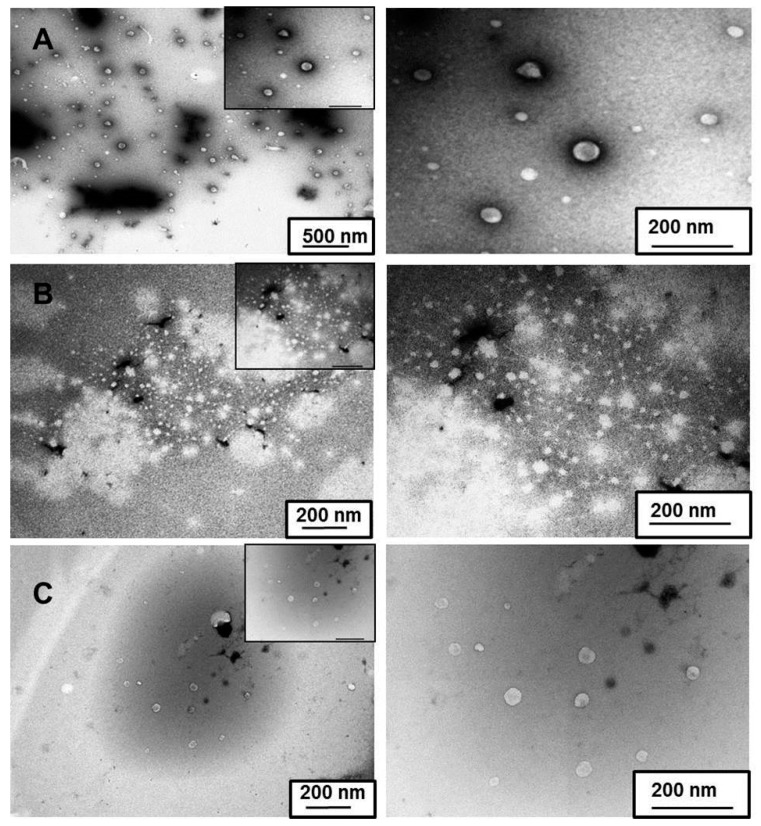
Transmission electron microscope (TEM) images of (**a**) F2 magnified by 15 k (left) and 60 k (right), (**b**) F3 magnified by 30 k (left) and 60 k (right) and (**c**) F5 magnified by 30 k (left) and 60 k (right).

Formulations F2, F3 and F5 were selected for morphology characterization by TEM (Figure 7).

In all formulations, aggregate particles were spherical or irregularly shaped. In the case of F2 (Figure 7a), aggregates were detected with varying sizes up to 500 nm. Smaller aggregates were found in F3 (Figure 7b) and F5 (Figure 7c), ranging in size between 200 and 300 nm. Size data obtained by TEM confirmed results regarding the size distribution of these nanoaggregates obtained through dynamic light scattering.

## 4. Conclusions

This study was the first to investigate the strategy of adding hydrophilic polymers to nepafenac drug/CD complexes that included two different CDs in order to improve drug solubility and promote nanoaggregate formation. Results show that HPβCD performed best in terms of solubilization, while γCD performed best in terms of enhancing nanoaggregate formation. The mean size of these aggregates was found in the range of 220–322 nm. Formation of inclusion complexes was confirmed by DSC, FT-IR and ^1^H-NMR studies. DSC studies suggested that at least 8% (*w*/*v*) HPβCD was needed for optimal complex formation when used with 15% (*w*/*v*) γCD. No synergistic effect on solubility was found using mixtures of γCD and HPβCD. Addition of hydrophilic polymers, in particular CMC, PVA and tyloxapol, to formulations containing CDs led to higher nepafenac solubility. Overall, results of this study on the solubility and aggregate formation associated with various CDs in polymer solutions indicates that formulations of polymeric drug/CD nanoaggregates can be formed easily and represent a promising new approach to the formulation of nepafenac eye drops.

## Figures and Tables

**Table 1:** Polymer composition of the nine eye drop formulations.

Formulations	PVP (% *w*/*v*)	PVA (% *w*/*v*)	CMC (% *w*/*v*)	HPMC (% *w*/*v*)	MC (% *w*/*v*)	Tyloxapol (% *w*/*v*)
F1	-	2.0	-	0.1	-	0.1
F2	-	-	1.0	-	-	-
F3	-	2.0	1.0	-	-	0.1
F4	-	2.0	-	-	-	0.1
F5	1.0	-	1.0	0.1	-	-
F6	1.0	-	-	-	0.1	-
F7	-	2.0	1.0	-	0.1	0.1
F8	1.0	-	-	-	-	0.1
F9	-	2.0	1.0	-	0.1	-

PVP, polyvinylpyrrolidone; PVA, hydrolysed poly(vinyl alcohol); CMC, carboxymethylcellulose; HPMC, hydroxypropylmethylcellulose; MC, methyl cellulose.

**Table 2:** Nepafenac concentrations in aqueous solution containing 1% *w*/*v* γCD after heating by sonication. Results are expressed as mean ± SD (n = 3).

Sonication	Mean (± SD) Nepafenac Concentration (µg/mL)
60 °C 20 min	6.41 ± 0.08
60 °C 40 min	6.49 ± 0.07
60 °C 60 min	6.57 ± 0.08

**Table 3:** Values of the apparent stability constant (K_1:1_) and complexation efficiency (CE).

Cyclodextrin	Type	Slope	Corr.	K_1:1_ (M^−1^)	CE	Solubility (mg/mL) in the Presence of 15% (*w*/*v*) CD
γCD	A_l_^a^	0.024	0.998	248	0.024	0.715
HPγCD	A_p_^a^	0.022	0.978	218	0.021	0.590
αCD	A_l_^a^	0.029	0.991	289	0.028	0.131
HPαCD	A_l_^a^	0.011	0.984	113	0.011	0.401
βCD	A_l_	0.180	0.998	2230	0.220	^b^
HPβCD	A_l_^a^	0.198	0.999	2515	0.247	4.460

Corr., Correlation; HP, 2-hydroxypropyl; ^a^ Measured from 0–15% CD; ^b^ βCD was not soluble in water at this concentration.

**Table 4:** H-NMR Chemical shift corresponding to free γCD alone and in the presence of nepafenac.

Protons	γCD	Nepafenac/γCD	Δδ*
H1	5.1320	5.1554	+0.0234
H2	3.6754	3.7031	+0.0277
H3	3.9564	3.9782	+0.0218
H4	3.6115	3.6339	+0.0224
H5	3.8712	3.8925	+0.0213
H6	3.8903	3.9146	+0.0243

Δδ* = δ_complex_ − δ_free_.

**Table 5:** H-NMR Chemical shift corresponding to free HPβCD alone and in the presence of nepafenac.

Protons	HPβCD	Nepafenac/HPβCD	Δδ*
H1	5.1207	5.1137	−0.007
H2	3.6686	3.6625	−0.0061
H3	4.0386	4.0046	−0.034
H4	3.5485	3.5468	−0.0017
H5	3.9010	3.7681	−0.1329
H6	3.9487	3.8912	−0.0575
–CH3	1.1952	1.1864	−0.0088

Δδ* = δ_complex_ − δ_free_.

**Table 6:** Characteristics of 0.5% (*w*/*v*) eye drop formulations including solubility of nepafenac, proportion of solid drug fraction, osmolality, viscosity and aggregate size.

Formulations	Solubility of Nepafenac (mg/mL)	Solid Drug Fraction (%)	Osmolality (mOsm/kg)	Viscosity (cP)	Size (nm)	
					Diameter (nm)	Vol (%)
F1 (2% PVA, 0.1 HPMC, 0.1% tyloxapol)	3.063 ± 0.108	61.26	198 ± 2	3.62 ± 0.03	98	58.1
					424	41.9
F2 (1% CMC)	2.516 ± 0.014	50.32	338 ± 13	18.92 ± 2.16	212	55.4
					135	28.9
					427	13.7
					18	2
F3 (2% PVA, 1% CMC, 0.1% tyloxapol)	3.180 ± 0.066	63.60	410 ± 10	13.95 ± 0.36	247	88.5
					3.0	11.5
F4 (2% PVA, 0.1% tyloxapol)	3.119 ± 0.010	62.38	200 ± 2	4.17 ± 0.11	581	29
					241	28.8
					1127	23.4
					106	18.8
F5 (1% PVP, 1% CMC, 0.1% HPMC)	2.656 ± 0.074	53.12	400 ± 8	15.31 ± 0.88	310	50.1
					170	27.5
					13	22.4
F6 (1% PVP, 0.1% MC)	2.384 ± 0.172	47.08	186 ± 5	4.89 ± 0.31	350	77.4
					21	22.6
F7 (2% PVA, 1% CMC, 0.1% MC, 0.1% tyloxapol)	2.817 ± 0.015	56.34	390 ± 4	10.15 ± 0.47	208	89.4
					644	9.1
					54	1.5
F8 (1% PVP, 0.1% tyloxapol)	1.685 ± 0.054	33.70	193 ± 2	3.83 ± 0.16	17	74.5
					781	22.5
					1.0	3
F9 (2% PVA, 1% CMC, 0.1% MC)	2.422 ± 0.056	48.44	392 ± 7	13.56 ± 0.92	208	97.7
					28	2.3

## Supplementary Materials

The following are available online at http://www.mdpi.com/1996-1944/12/2/229/s1, Figure S1: Chemical structure of (a) natural cyclodextrins (α-, β- and γ-CDs) and (b) their hydroxyl-propyl derivates (HPα-, HPβ- and HPγ-CDs), Figure S2: H-NMR spectra of (a) HPβCD alone (top) and their complex (bottom) and (b) γCD alone (top) and their complex (bottom), Table S1: Effect of cyclodextrins and excipients on osmolality, viscosity and size of binary and ternary systems with nepafenac, Table S2: Chemical shift corresponding to free nepafenac alone and in the presence of γCD or HPβCD.
