# Supplementary material for: Nepafenac-Loaded Cyclodextrin/Polymer Nanoaggregates: A New Approach to Eye Drop Formulation
Source: Materials (Basel). 2019 Jan 11;12(2):229. doi: 10.3390/ma12020229 (PMC6356765; doi:10.3390/ma12020229)
Supplement: Supplementary file 1 [file materials-12-00229-s001.pdf]

materials

Supplementary Information

Nepafenac-Loaded Cyclodextrin/Polymer Nanoaggregates: A New Approach to Eye Drop Formulation

Blanca Lorenzo-Veiga, Hakon Hrafn Sigurdsson * and Thorsteinn Loftsson

Faculty of Pharmaceutical Sciences, University of Iceland, Hofsvallagata 53, IS-107 Reykjavik, Iceland; blv3@hi.is (B.L.-V.); thorstlo@hi.is (T.L.)

* Correspondence: hhs@hi.is; Tel.: +354-525-5821

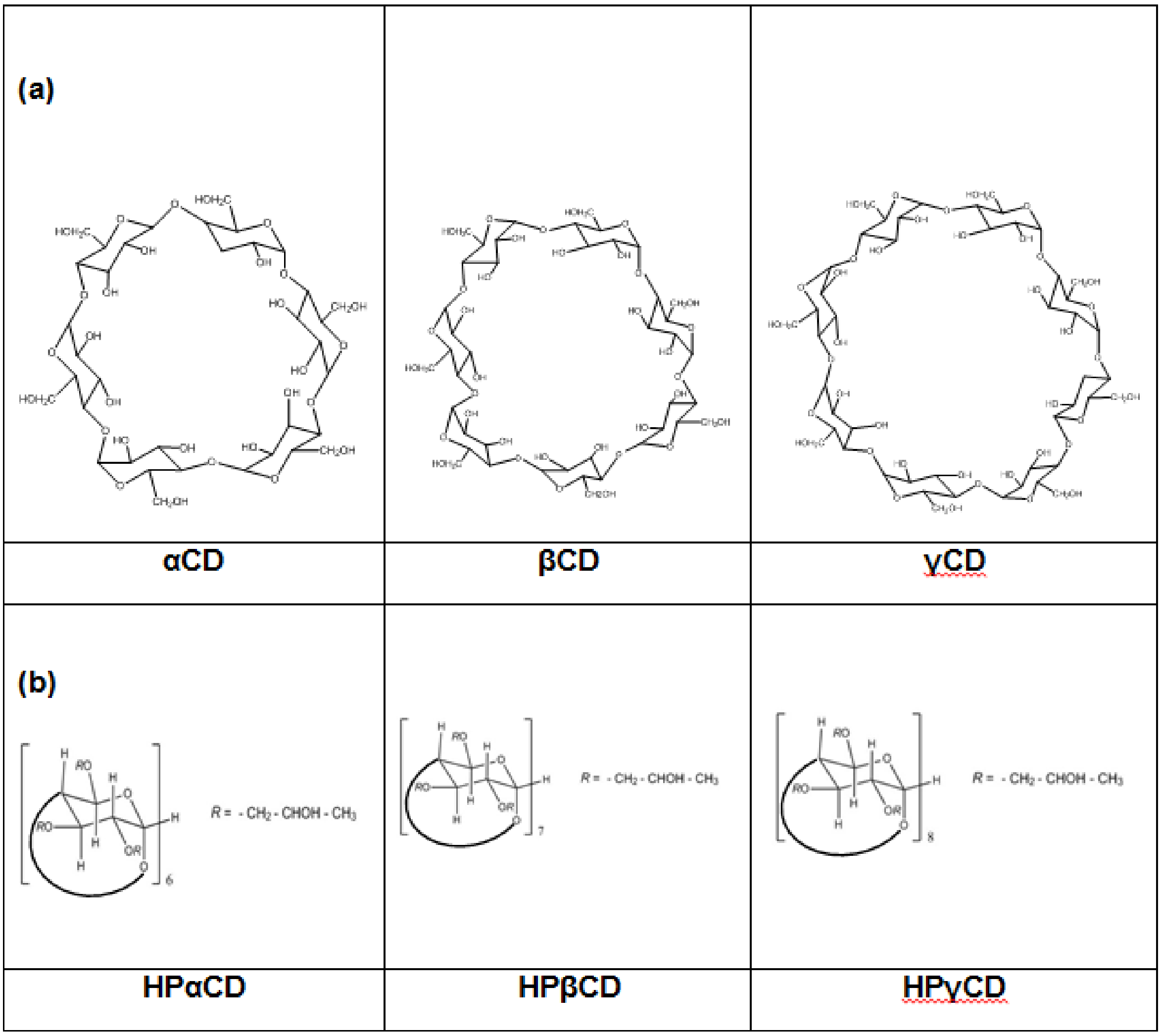

Figure S1. Chemical structure of (**a**) natural cyclodextrins (α-, β- and γ-CDs) and (**b**) their hydroxylpropyl derivates (HPα-, HPβ- and HPγ-CDs).

(a)

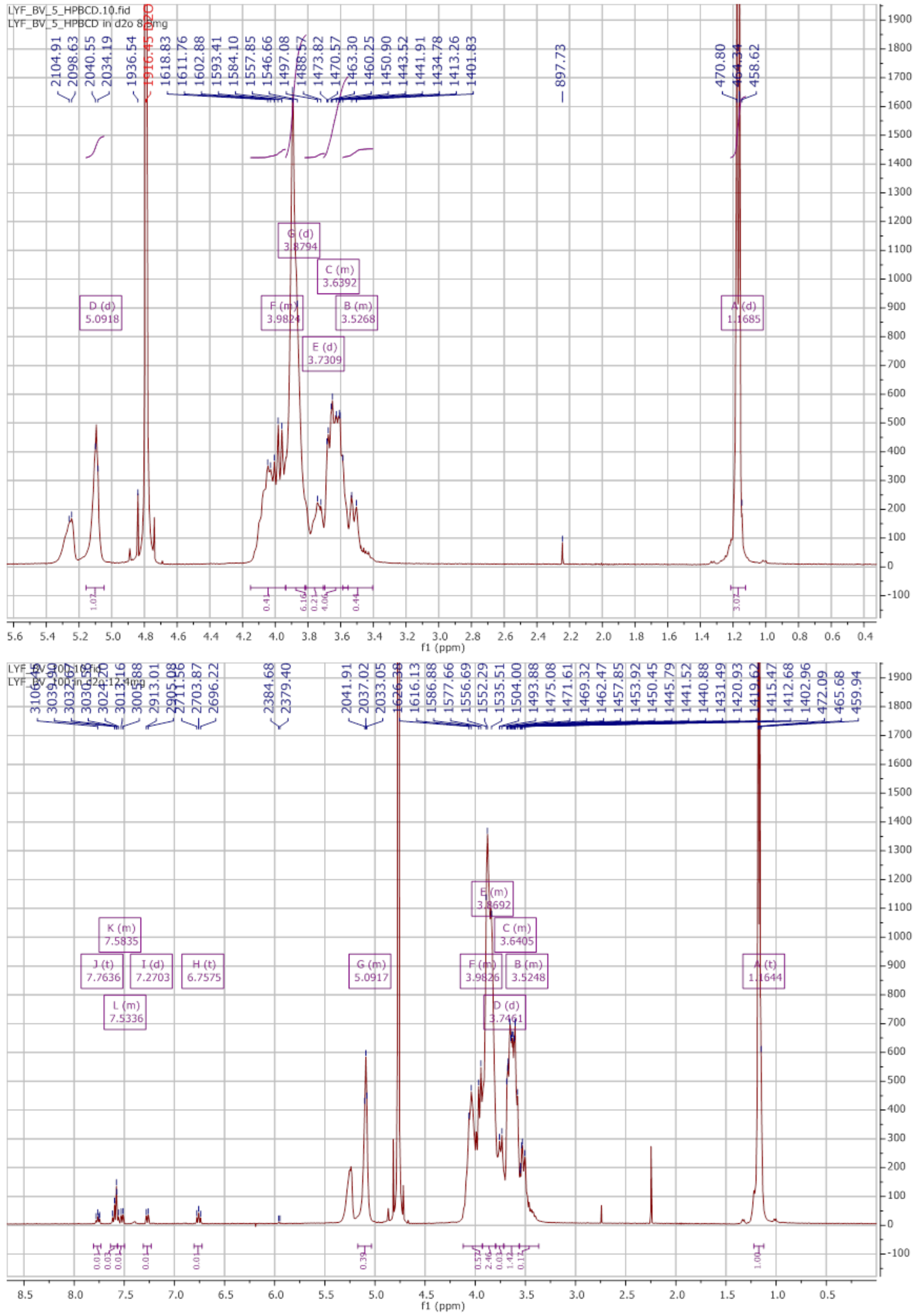
LYF_BV_5_HPBCD.10.fid
LYF_BV_5_HPBCD in d2o 8.3mg
D (d) 5.0918
G (d) 3.8794
C (m) 3.6392
F (m) 3.9824
B (m) 3.5268
E (d) 3.7309
A (d) 1.1685
f1 (ppm)
K (m) 7.5835
J (t) 7.7636
I (d) 7.2703
H (t) 6.7575
L (m) 7.5336
G (m) 5.0917
E (m) 3.8692
C (m) 3.6405
F (m) 3.9826
B (m) 3.5248
D (d) 3.7461
A (t) 1.1644
f1 (ppm)

(b)

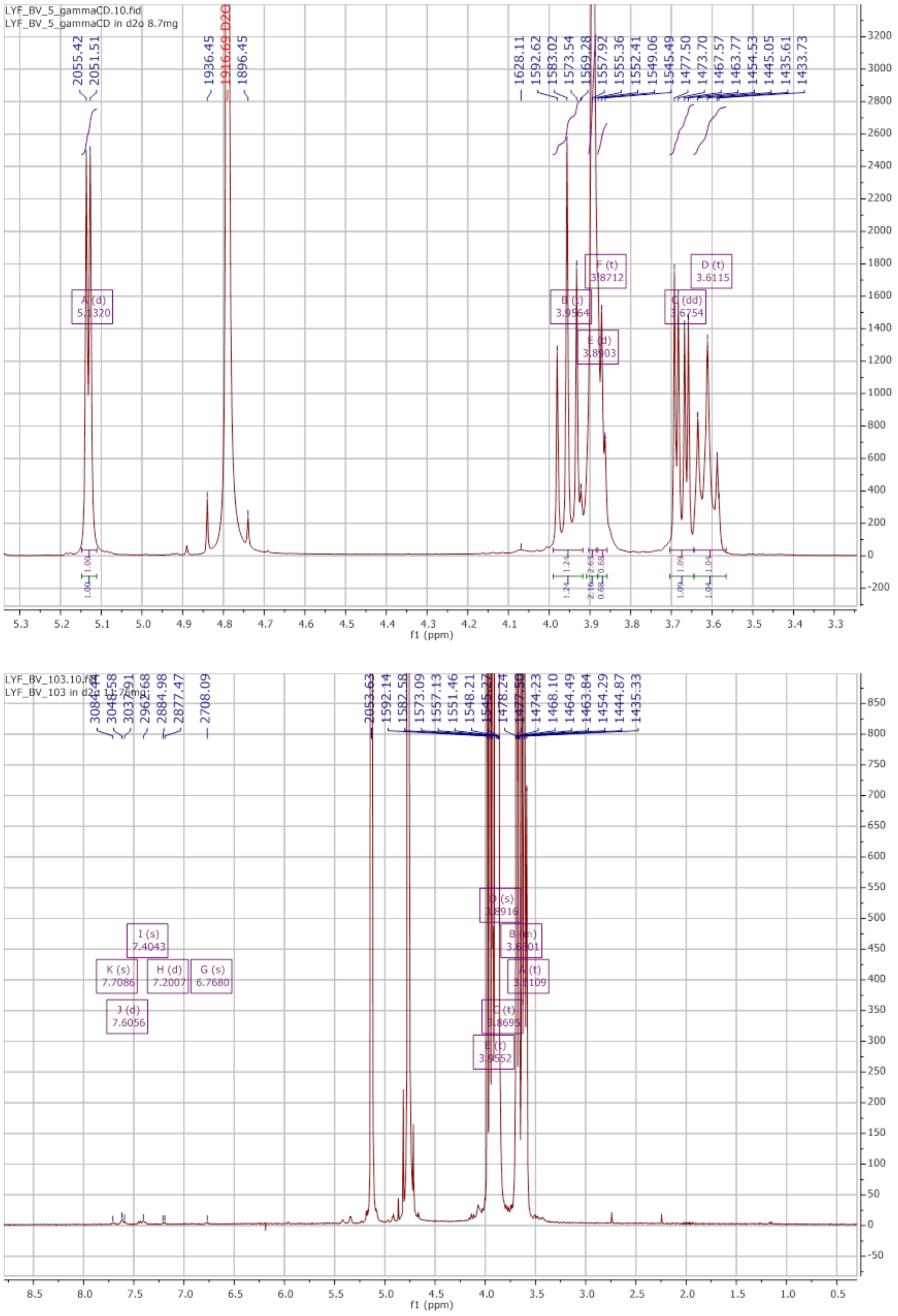

Figure S2. H-NMR spectra of (**a**) HPβCD alone (top) and their complex (bottom), and (**b**) γCD alone (top) and their complex (bottom).

Table S1. Effect of cyclodextrins and excipients on osmolality, viscosity and size of binary and ternary systems with nepafenac.

Cyclodextrin	Excipient	Osmolality (mOsm/kg)	Viscosity (cP)	Size (nm)*
15%γCD	No additive	120 ± 0	1.89 ± 0.38	220.3
	PVA	140 ± 1	3.20 ± 0.02	296.1
	PVP	161 ± 1	3.22 ± 0.04	163.6
	Tyloxapol	117 ± 2	2.22 ± 0.02	230.7
	CMC	166 ± 1	4.34 ± 0.23	322
15%γCD/2.5%HPβCD	No additive	71 ± 1	1.40 ± 0.06	277.8
	PVA	117 ± 1	2.89 ± 0.06	279.2
	PVP	125 ± 2	1.93 ± 0.02	281.7
	Tyloxapol	102 ± 2	1.69 ± 0.02	317
	CMC	133 ± 3	6.92 ± 0.1	317
15%γCD/ 5%HPβCD	No additive	88 ± 2	1.54 ± 0.02	180.8
	PVA	158 ± 1	3.16 ± 0.01	547
	PVP	149 ± 0	2.02 ± 0.10	306
	Tyloxapol	130 ± 2	1.83 ± 0.02	1466
	CMC	166 ± 2	11.00 ± 0.10	459
15%γCD/ 8%HPβCD	No additive	101 ± 2	1.53 ± 0.02	161.2
	PVA	219 ± 1	4.37 ± 0.05	246.5
	PVP	220 ± 2	2.37 ± 0.06	1524
	Tyloxapol	204 ± 2	2.37 ± 0.02	730
	CMC	238 ± 2	16.00 ± 0	319

* The diameter of the mean population (nm) is displayed.

Table S2. Chemical shift corresponding to free nepafenac alone and in the presence of γCD or HPβCD.

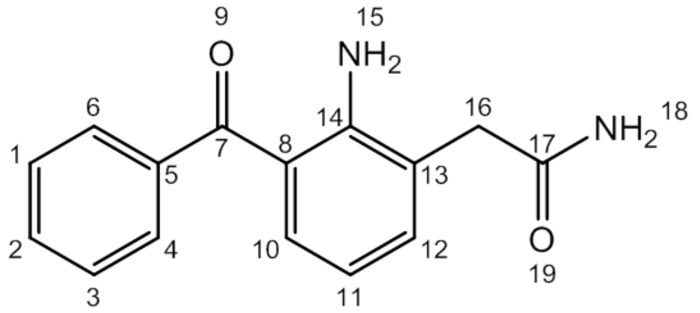

Protons	Nepafenac	Nepafenac/HPβCD	Δδ*	Nepafenac	Nepafenac/γCD	Δδ*
H-4	7.6301	7.7301	0.1	7.6301	7.7856	0.1555
H-6	7.6301	7.7316	0.1015	7.6301	7.7856	0.1555
H-2	7.5236	7.6286	0.105	7.5236	7.6055	0.0819
H-3	7.4513	7.4273	−0.024	7.4513	7.5556	0.1043
H-1	7.4513	7.4273	−0.024	7.4513	7.5556	0.1043
H-12	7.2373	7.2237	−0.0136	7.2373	7.2973	0.06
H-11	6.8189	6.7910	−0.0279	6.8189	6.7793	−0.0396
H-16	3.5635	*	*	3.5635	*	*

* Due to the overlapping of CD protons, it is not possible to access the resonance of proton.
